# Acute Respiratory Failure and Metabolic Acidosis Due to Excessive Normal Saline Absorption During Hysteroscopic Myomectomy: A Case Report

**DOI:** 10.7759/cureus.86591

**Published:** 2025-06-23

**Authors:** Haruka Naiki, Shinji Sugita

**Affiliations:** 1 Department of Anesthesiology, Nippon Medical School Musashi-Kosugi Hospital, Kawasaki, JPN

**Keywords:** acidosis, case report, fluid overload, hysteroscopy, normal saline, pulmonary edema

## Abstract

Hysteroscopic myomectomy commonly employs fluid irrigation to maintain visualization of the uterine cavity, with normal saline often used as the distension medium due to its favorable safety profile. However, significant intravascular absorption of isotonic fluids can result in life-threatening complications. We report a case of acute respiratory failure and severe hyperchloremic metabolic acidosis caused by excessive absorption of normal saline during hysteroscopic myomectomy. The patient required mechanical ventilation and intensive care but recovered fully with timely and appropriate management. This case underscores the need for vigilant fluid balance monitoring during hysteroscopic procedures, even when isotonic irrigation solutions are used.

## Introduction

Hysteroscopic surgery has become a cornerstone procedure for managing intrauterine pathologies, including submucosal fibroids and endometrial polyps. Its minimally invasive nature offers reduced recovery times and lower complication rates compared to open surgical approaches. A key component of hysteroscopic techniques is the use of continuous fluid irrigation to distend the uterine cavity and maintain adequate visualization [1,2].

Although hypotonic distension media, such as glycine and sorbitol, have historically been associated with risks like dilutional hyponatremia, cerebral edema, seizures, and pulmonary edema resulting from fluid overload [3], isotonic solutions, such as normal saline, are often preferred for their more favorable safety profile. However, significant intravascular absorption of isotonic fluids can result in serious complications, including pulmonary edema and metabolic derangements. This highlights that isotonicity does not eliminate the risk of fluid-related adverse events.

The incidence of fluid overload during operative hysteroscopy is reported to range from 0% to 5%, with contributing risk factors including prolonged operative time, large lesion size, and elevated infusion pressures [4,5]. Although most documented complications, such as water intoxication, involve hypotonic media, severe pulmonary events associated with isotonic normal saline irrigation are less frequently reported. We present a case illustrating the potential for normal saline to cause acute respiratory failure and severe hyperchloremic metabolic acidosis when absorbed in excessive amounts beyond physiological compensation.

## Case presentation

A 39-year-old woman (height: 150.8 cm, weight: 42.5 kg) with no significant medical history was admitted for resection of a 40-mm submucosal uterine myoma, identified during infertility treatment.

Hysteroscopic myomectomy for a submucosal myoma located on the posterior wall of the uterus (International Federation of Gynecology and Obstetrics (FIGO) Leiomyoma Subclassification System: Type 2) was performed under combined spinal and general anesthesia [6]. The patient was informed about the possibility of a two-stage surgery. Initially, spinal anesthesia was administered using 3 mL of 0.5% isobaric bupivacaine at the L3/4 level, achieving analgesia up to the level of the 10th thoracic vertebra within 10 minutes. Continuous intravenous propofol infusion was then administered at a plasma target concentration of 3.0 μg/mL using a target-controlled infusion pump (Terumo, Tokyo, Japan). Oxygen was delivered via a face mask at 6 L/minute during the surgery.

Sixty minutes into the procedure, the patient developed involuntary tachypnea and hypotension. The amount of normal saline irrigation used had not been recorded. For hypotension, phenylephrine, a vasopressor, was administered. Suspecting possible uterine perforation, insufficient anesthetic effect, or other complications, oxygen saturation declined progressively from 98% to 92%. Urgent airway management was initiated using a supraglottic airway device (i-gel®, Intersurgical, Wokingham, UK), and general anesthesia was induced. Anesthesia was maintained with 2 vol% sevoflurane in 3 L/minute of a 50% oxygen and 50% nitrous oxide mixture (fraction of inspired oxygen = 0.5), along with 5 cmH_2_O of positive end-expiratory pressure (PEEP).

**Figure 1:**
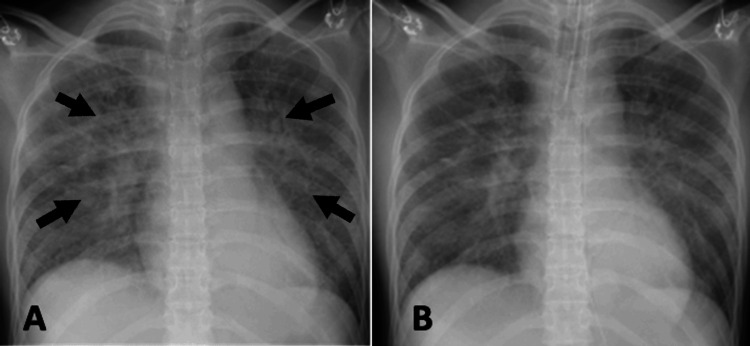
Intraoperative chest radiographs (A) Before endotracheal intubation: Pulmonary edema is visible (arrows), likely caused by excessive absorption of saline. (B) After endotracheal intubation: Pulmonary edema shows slight improvement following the application of positive end-expiratory pressure.

Arterial blood gas (ABG) analysis revealed mild respiratory failure (PaO_2_/FiO_2_ (P/F) ratio = 224) and severe metabolic acidosis (pH = 7.165), with marked hyperchloremia (chloride = 125 mEq/L) (Table 1). Chest radiography confirmed pulmonary edema; however, oxygenation did not improve. Endotracheal intubation was subsequently performed to ensure adequate PEEP (Figure 1).

**Table 1:** Perioperative arterial blood gas analysis BE: base excess; Cl: chloride; FiO₂: fraction of inspired oxygen; HCO₃: bicarbonate; K: potassium; Na: sodium; pCO₂: partial pressure of carbon dioxide; PEEP: positive end-expiratory pressure; pH: potential of hydrogen; pO₂: partial pressure of oxygen

	Initiation of intubation (FiO_2_ = 0.5, PEEP = 5 cmH_2_O)	One hour after surgery (FiO_2_ = 0.5, PEEP = 8 cmH_2_O)	12 hours after surgery (FiO_2_ = 0.5, PEEP = 8 cmH_2_O)	24 hours after surgery (FiO_2_ = 0.21, without PEEP)
pH	7.165	7.374	7.518	7.444
pCO_2 _(mmHg)	44.8	33.5	29.8	37
pO_2 _(mmHg)	112	105	283	86
HCO_3 _(mEq/L)	15.5	19.5	24.9	24.9
BE (mEq/L)	-12	-5.0	1.8	1.4
Na (mEq/L)	139	149	142	138
K (mEq/L)	3.1	2.3	2.7	3
Cl (mEq/L)	125	125	111	109
Lactate (mmol/L)	0.4	0.7	0.8	1.1

The procedure was discontinued after hemostasis was achieved, with a net positive fluid balance of +7,650 mL (total input: 17,300 mL; output via outflow tract and urine: 9,650 mL). The amount of hemorrhage was unknown because it was contained within the drainage of the irrigation fluid. Acute cardiac failure was ruled out via transthoracic echocardiography. The surgery lasted 91 minutes, and the total anesthesia time was 212 minutes. The volume of intravenous administration was 1,050 mL.

**Figure 2:**
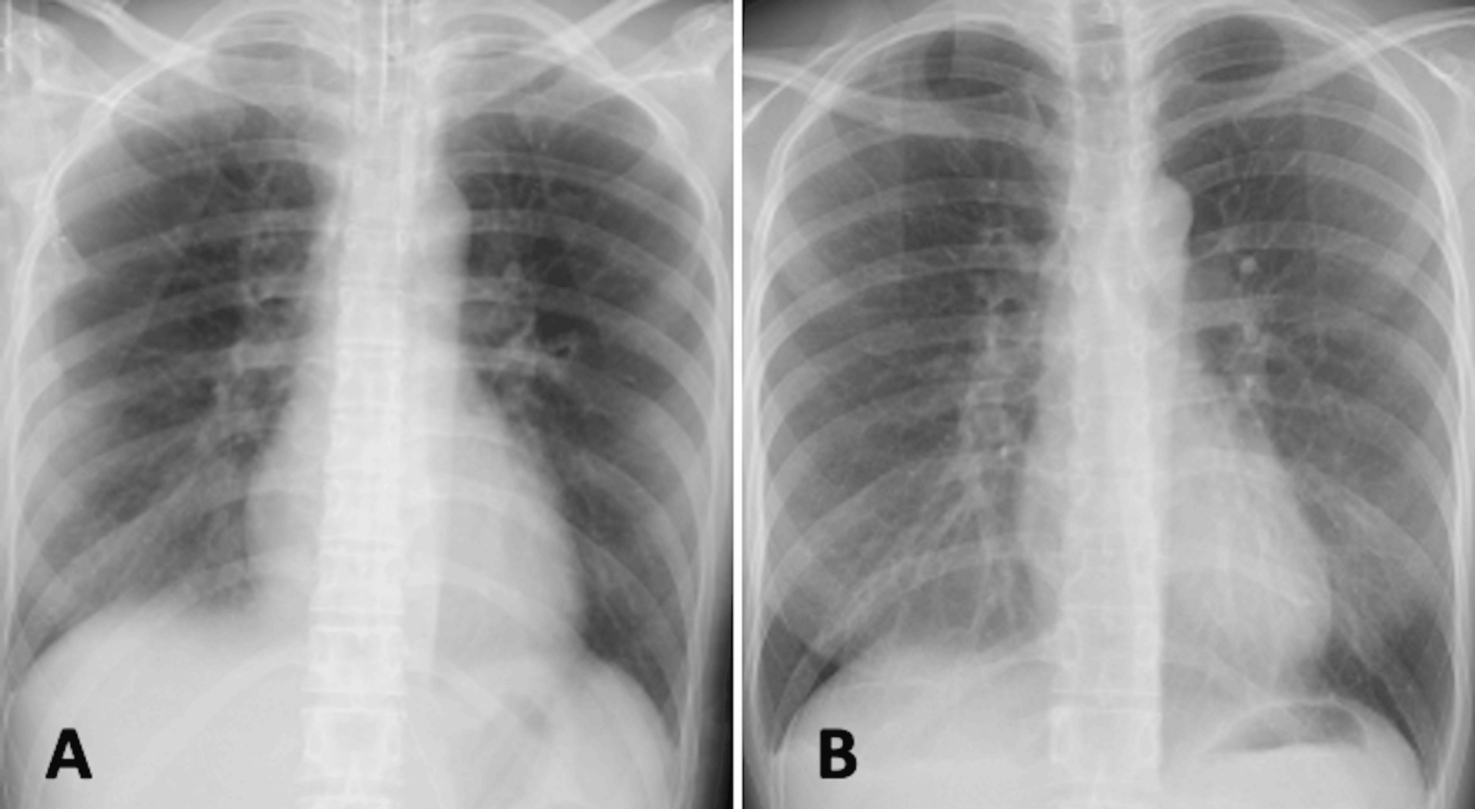
Postoperative chest radiographs (A) Postoperative day (POD) 1: Pulmonary edema markedly improved following mechanical ventilation and diuretic therapy. (B) POD 4: Pulmonary findings nearly normalized.

The patient was transferred to the intensive care unit (ICU), where mechanical ventilation with 8 cmH2O of PEEP was initiated. Intravenous furosemide was administered to promote diuresis. The patient’s oxygenation status and ABG values gradually improved. She was safely extubated after 12 hours and transferred to the general ward the same day. She was discharged on postoperative day 7 without sequelae (Figure 2) and was scheduled for two-stage surgery.

## Discussion

In the present case, the patient developed acute respiratory failure and severe hyperchloremic metabolic acidosis following hysteroscopic myomectomy, during which normal saline was used as the irrigation fluid. Although normal saline is generally regarded as a safe isotonic medium, excessive intravascular absorption during hysteroscopic procedures can lead to life-threatening complications.

The American Association of Gynecologic Laparoscopists guidelines emphasize the importance of monitoring fluid balance and establishing safe fluid deficit limits during hysteroscopic surgery [1]. Chung et al. reported that a myoma diameter of ≥4.0 cm is an optimal threshold for predicting the use of more than 5,000 mL of distension fluid during hysteroscopic myomectomy [7]. In our case, the high volume of absorbed fluid likely exceeded the patient's pulmonary capillary filtration capacity, resulting in pulmonary edema. This was further complicated by significant hyperchloremic metabolic acidosis, which contributed to respiratory failure and hemodynamic instability. Although water intoxication syndrome is most commonly associated with the absorption of hypotonic distension media, such as 1.5% glycine, its clinical features overlap with those observed in this case. Water intoxication typically presents with respiratory distress, airway edema, pulmonary edema, metabolic acidosis, hyponatremia, hypokalemia, and circulatory collapse [3,8]. In our patient, several of these manifestations were observed, including respiratory distress, mild airway edema, marked pulmonary edema, hyperchloremic metabolic acidosis, hypokalemia, and mild circulatory instability; however, hyponatremia was absent. Prough and Bidani demonstrated that hyperchloremic metabolic acidosis is a predictable, dose-dependent consequence of saline infusion, supporting the pathophysiological mechanisms observed in our patient [9]. Technical factors may further increase the risk of unrecognized fluid overload. Boyd and Stanley identified common errors in fluid tracking during hysteroscopy, which may lead to underestimation of fluid absorption [10]. These factors underscore the importance of rigorous intraoperative monitoring.

Several factors may contribute to the development and delayed recognition of hyperchloremic metabolic acidosis and fluid overload during hysteroscopic procedures. As summarized in Table 2, these include patient-specific conditions such as hypotension and respiratory compensation, environmental factors such as inadequate fluid balance monitoring, and technical aspects of intraoperative care. For instance, hyperchloremic metabolic acidosis, although often considered benign, has been shown to impair arterial pressure [11]. Similarly, continuous oxygen administration may mask compensatory tachypnea, delaying the recognition of respiratory decompensation. In addition, relying solely on the anesthesiologist’s discretion to perform ABG analysis may result in missed metabolic disturbances. Regular interval ABG testing or the use of lung assessment tools, such as lung ultrasound, may facilitate earlier detection of pulmonary congestion [12]. Moreover, a key issue was the lack of clearly defined criteria for discontinuing the procedure when resection became difficult. In this case, it was assumed from the outset that two-stage surgery might be necessary. Therefore, it is essential to plan a comprehensive perioperative strategy that includes the possibility of staging the procedure.

**Table 2:** Main predictable factors and clinical indicators of excessive normal saline absorption during hysteroscopy

Category	Factor/indicator	Comment
Procedure-related	Large myoma size	Associated with higher irrigation fluid use and increases cumulative risk of fluid absorption.
	The use of high volume of distension fluid	
	Prolonged operative time	
Patient-related	Hypotension	Indicative of pulmonary edema or metabolic acidosis.
	Worsening of respiratory status	
Environmental/technical	Inadequate monitoring of fluid input/output	Delays detection of significant fluid absorption.
	Continuous oxygen therapy	Delays recognition of respiratory failure.
	Absent arterial blood gas analysis	Misses impaired oxygenation or metabolic derangements.
	Lack of screening tools (e.g., lung ultrasound)	Misses signs of fluid overload.

Respiratory management strategies for fluid overload vary. Kim et al. described a similar case in which intraoperative respiratory failure occurred while using an i-gel airway; oxygenation improved upon termination of anesthesia and initiation of noninvasive positive pressure ventilation [13]. Similarly, Summers et al. reported successful treatment of acute pulmonary edema following hysteroscopy using continuous positive airway pressure [14]. In our case, chest radiographs during the procedure revealed significant pulmonary edema, prompting immediate tracheal intubation and mechanical ventilation in the ICU. Although respiratory failure secondary to fluid overload is largely volume-dependent, individualized evaluation and intervention remain critical.

The standard treatment approach involves diuresis, positive pressure ventilation, and PEEP. Neurological complications, such as fluid and electrolyte imbalances, vision disturbances, confusion, and in severe cases, coma or even death, have been associated with pulmonary edema due to absorption of hypotonic fluids such as 1.5% glycine, often requiring prolonged treatment [15,16]. In contrast, complications arising from normal saline tend to be more responsive to treatment and are associated with a relatively favorable prognosis.

## Conclusions

This case illustrates that even isotonic distension media, such as normal saline, can lead to serious complications during hysteroscopic procedures when used in excessive volumes. Pulmonary edema and hyperchloremic metabolic acidosis may develop rapidly and require intensive respiratory and metabolic support. Although normal saline is generally considered safe, its risk should not be underestimated in the setting of high intravascular absorption. Careful intraoperative monitoring, early recognition of fluid overload, and coordinated management by surgical and anesthetic teams are essential for ensuring patient safety.
